# Hypertension a Predictive Risk Factor on Progression to Alzheimer’s Disease Using APOEε4 as a Benchmark

**DOI:** 10.3390/brainsci15050434

**Published:** 2025-04-23

**Authors:** Mingfei Li, Ying Wang, Lewis Kazis, Weiming Xia

**Affiliations:** 1 Department of Mathematical Sciences, Bentley University, Waltham, MA 02452, USA; 2 Center for Healthcare Organization and Implementation Research, Bedford VA Healthcare System, Bedford, MA 01730, USA; 3 Geriatric Research Education and Clinical Center, Bedford VA Healthcare System, Bedford, MA 01730, USA; wangy14@wit.edu; 4 School of Computing and Data Science, Wentworth Institute of Technology, Boston, MA 02115, USA; 5 Department of Health Law, Policy and Management, Boston University School of Public Health, Boston, MA 02118, USA; lek@bu.edu; 6 Rehabilitation Outcomes Center (ROC), Spaulding Rehabilitation Hospital, Harvard Medical School, Boston, MA 02115, USA; 7 Department of Biological Sciences, University of Massachusetts, Lowell, MA 01854, USA; 8 Department of Pharmacology, Physiology & Biophysics, Boston University Chobanian & Avedisian School of Medicine, Boston, MA 02118, USA

**Keywords:** Alzheimer’s disease, mild cognitive impairment, hypertension, hypercholesterolemia, APOE

## Abstract

**Background:** Comorbidities such as hypertension and hypercholesterolemia are risk factors associated with Mild Cognitive Impairment (MCI) and Alzheimer’s disease (AD). The most significant genetic risk factor is the ε4 allele of the apolipoprotein E gene (APOE). The aim of this paper is to determine whether hypertension is the most significant but modifiable risk factor to delay AD onset. **Method:** A cohort of patients with MCI (N = 3052) is developed from the documented database (N = 43,999) within the National Alzheimer’s Coordinating Center (NACC) during the time period from June 2005 to May 2021. Cox proportional hazard models with propensity score weights on demographic information and comorbidities at baseline are applied to examine association of hypertension and hypercholesterolemia with AD onset among MCI patients. Associations are compared to APOE genotypes and AD onset. In addition, the association of hypertension with decline rates in Mini-Mental State Examination (MMSE) scores are reported. **Results:** After controlling for age, sex, race, APOEε4, and reported comorbidities, the results show that MCI patients who subsequently develop hypertension within 18 months after their first diagnosis of MCI have a significantly higher risk of AD onset (HR = 2.77, 95%CI (1.66, 4.65), *p* value < 0.0001), compared to MCI patients with no hypertension or a late occurrence of hypertension after 18 months. This significant association is validated through a Random Forest method, a machine learning approach with bootstrap simulations. In addition, patients with early hypertension have significantly higher MMSE score declining rates compared to those without hypertension (coefficient = 0.988, *p* = 0.0054.). **Conclusions:** Hypertension is the most significant risk factor comparable to the genetic risk factor APOEε4 allele. Our finding is unique, as we did not observe a similar outcome in those with early hypercholesterolemia. Thus, among all comorbidities, hypertension is the most significant risk factor similar to the genetic risk factor APOEε4 allele.

## 1. Introduction

A systematic review published in 2023 reported a global prevalence rate of Mild Cognitive Impairment (MCI) of 19.7%, and this rate has significantly increased over time since 2019 [1]. A considerable proportion of individuals with MCI progress to Alzheimer’s Disease (AD) with an annual conversion rate of 10–15% [2]. In addition, chronic disease prevalence is strikingly high in the United States, with the Centers for Disease Control and Prevention (CDC) reporting that nearly half of the adult population (119 million) have hypertension as of 2020 [3].

The relationship between chronic diseases such as hypertension, hypercholesterolemia, diabetes, and cognitive impairment has garnered increased attention in recent years. A previous study found an association between long-term hypertension from mid- to late-life and an increased risk of dementia [4]. Another meta-analysis study provided a comprehensive overview of the relationship between blood pressure and cognitive decline [5]. Using mortality as a competing risk factor, a recent study also examined the relationship between blood pressure and dementia risk [6]. Similarly, hypercholesterolemia has been a focus of interest in those with cognitive impairment with underlying mechanisms linking cholesterol to AD pathogenesis [7] and how hypercholesterolemia may contribute to AD progression [8]. Diabetes has been reported to be associated with an increased risk of dementia [9], and increased glycemic variability (increased glycated hemoglobin (HbA1c)) has been found to impact brain function in patients with progressive supranuclear palsy (PSP) or corticobasal syndrome (CBS) [10].

The potential benefits of angiotensin-converting enzyme (ACE) inhibitors and statins in AD prevention have been tested among patients with a history of traumatic brain injury [11]. By comparing the effectiveness of monotherapy versus combination therapies in AD treatment [12], recent studies suggest that angiotensin receptor blockers (ARBs) may play a significant role in delaying AD onset, lending support to the hypothesis that cardiovascular health is intrinsically linked to cognitive well-being. Other anti-hypertension drugs such as calcium channel blockers (e.g., nilvadipine) are reported to maintain cognitive function for 20 months among patients with MCI [13]. These cardiovascular drugs may reduce the incidence of AD following such injuries, collectively underscoring the complex interrelationships between cardiovascular health and cognitive function and highlighting potential avenues for AD prevention and management.

APOE is a gene with different alleles, each having a distinct impact on the risk and progression of AD [14]. Of these, the APOEε4 allele is the most notable, being the strongest genetic risk factor that is associated with AD. The APOEε2 allele is considered potentially protective of AD. The APOEε3 allele is not an AD risk factor and is the most common allele in the general population. Studies have shown that different APOE alleles can interact with other proteins and processes involved in Alzheimer’s pathology [15]. The impact of the APOEε4 allele on AD onset and progression have been well investigated and established [14].

This study focuses on the association of early hypertension (diagnosed within 18 months after the first MCI diagnosis) with AD onset among patients with MCI. We further compare early occurrence of hypertension to the genetic risk factor APOEε4 in influencing the progression from MCI to AD.

## 2. Methods

### 2.1. Study Population and Sample

Patient data with longitudinal medical history were obtained from the NACC database, covering 43,999 subjects between June 2015 and May 2021. Variables include patient clinical visit information, demographic information, cognitive test scores, genotypes, and comorbidities based on patient reported history of diagnosis.

**Figure 1:**
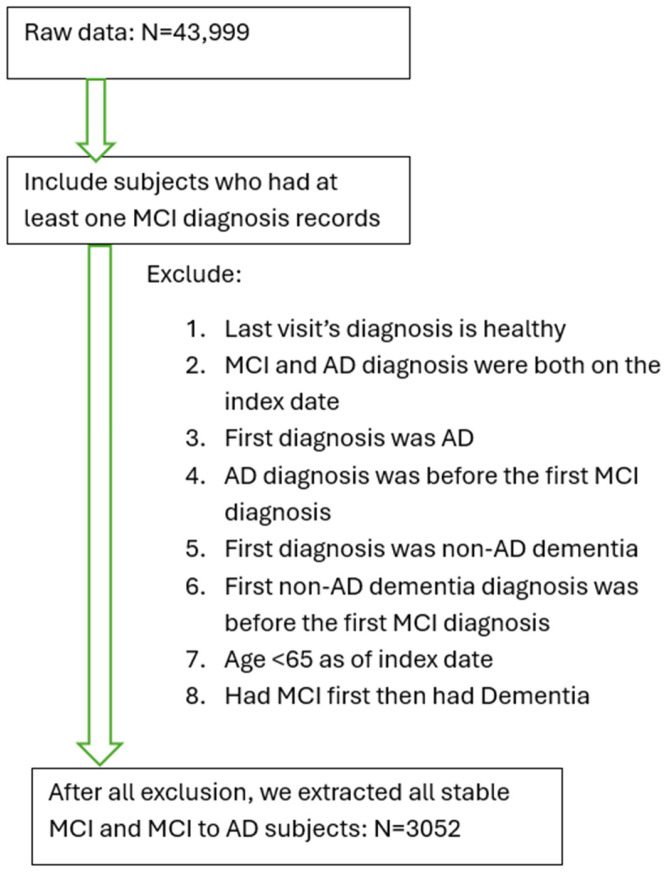
Inclusion and exclusion criteria to establish the cohort of patients with mild cognitive impairment (MCI) or AD converted from MCI.

Inclusion and exclusion criteria were as follows. A patient was identified as MCI if he/she had been diagnosed with Amnestic MCI or non-Amnestic MCI according to the diagnostic criteria [16]. If a patient’s record showed a diagnosis with any cognitive impairment (“dementia”, “MCI”, or “impaired, not MCI”) and at least two diagnoses of AD (to confirm no rule out diagnosis) after the first MCI diagnosis, this subject was defined as an AD patient (i.e., developed AD from the MCI stage). Patients diagnosed with MCI first and followed up with an AD diagnosis were defined as MCI-to-AD subjects (N = 1330). Additional criteria included age >= 65 by the end of the study (1 May 2021), and MCI diagnosis being given prior to the AD diagnosis or any dementia diagnosis. Patients whose first visit date included the diagnosis of AD or dementia were excluded from the study. Subjects with a normal cognitive test score (Mini Mental Status Exam (MMSE)) on record after their MCI diagnosis were excluded from the study. Based on our inclusion and exclusion criteria, we selected a cohort of 3052 qualified MCI patients, including 1330 MCI patients who converted to AD later (MCI-to-AD) (Figure 1). This study was approved by the Bedford VA Healthcare System Institutional Review Board and all data have been de-identified.

From a total of 43,999 subjects within the database of National Alzheimer’s Coordinating Center (NACC), 13,087 subjects were identified based on the diagnosis of MCI. Based on the exclusion criteria, a cohort of patients with MCI (stable MCI) or AD converted from MCI was established (N = 3052) (Table 1). Most subjects with MCI are at an advanced age (Supplement Table S1).

### 2.2. Early Hypertension, Early Hypercholesterolemia and Cognitive Decline Rate

To study the impact of early occurrence of hypertension on disease progression from MCI to AD, we examined patients’ self-reported conditions and excluded patients who had hypertension before their index date (the first date of MCI diagnosis). We defined early occurrence of hypertension (“early hypertension”) as the time from the index date to the first hypertension diagnosis date being less than 1.5 years (18 months), otherwise defined as “MCI without early hypertension”, including the patients who had no hypertension. Similarly, for the association of early occurrence of hypercholesterolemia (“early hypercholesterolemia”) with the AD onset among this MCI patient group, we excluded patients who had hypercholesterolemia before their index date. We defined early hypercholesterolemia as a time of less than 1.5 years from the first index date to the first hypercholesterolemia diagnosis date. We computed the survival time from the patient’s index date to first AD diagnosis date as the key outcome variable for the survival analyses.

In addition to the survival time to AD onset, we collected patients’ cognitive test scores (MMSE) [17] for all visits during the study period and computed the annual cognitive score declining rate (ACSDR) as:

$$annual\;cognitive\;score\;decline\;rate=\frac{\left(highest\;score-last\;visit\;score\right)}{\frac{last\;visit\;date-Index\;date}{365\;days}}$$

This ratio provides an estimation of the patient’s cognitive score declining rate during the study period, which is considered as an outcome measure of disease progression. For example, a subject’s highest MMSE score during the study period (from the index date to the last visit date) was 29. The subject’s score was 24 on the last visit day, which was 2 years later. The annual cognitive score decline rate (ACSDR) was (29 − 24)/2 = 2.5 per year, which indicates the patient’s MMSE score declines on average 2.5 per year after the index date assuming a linear decline. A subject with a higher ACSDR indicates that the MMSE score declines faster.

### 2.3. Analysis Methods

We reported the descriptive patient cohort statistics for comorbidities and outcome measures for the survival time from first MCI diagnosis to the first AD diagnosis including means, standard deviation and proportions. We utilized Cox proportional hazard models, controlling for demographic information, comorbidities, APOEε2/3/4 genotypes, and baseline cognitive test scores, to assess the risks that these conditions pose for AD development. We examined the associations of early hypertension and early hypercholesterolemia on AD progression in patients initially diagnosed with MCI. For comparison, we also utilized the unadjusted Cox model, adjusted Cox model, and propensity score weighted (PSW) Cox model to examine the association of early occurrence of hypertension and hypercholesterolemia. All hazard ratios (HR) are reported with 95% confidence intervals.

Propensity score weights for all models of both target chronic diseases were computed based on the demographic information, sex, age, race and APOE genetic information. We included the APOEε4 genetic indicator in the model as a covariate. The comorbidities, such as thyroid disease, heart attack/cardiac arrest, stroke, transient ischemic attack, alcohol abuse, B12 deficiency, congestive heart failure, seizures, and Parkinson’s Disease, were included in all the models to control for potential confounding effects. We also conducted a sensitivity analysis to verify our major findings from the statistical modeling in our data analysis. We applied the Survival Random Forest method [18], a machine learning method with simulation to assess the robustness of the models, to verify the impact of early onset of hypertension and hypercholesterolemia on the survival time to AD. The importance of variables and their associated significance are reported.

In addition, we employed Poisson mode with the study period as the offset to examine the association of early hypertension and early hypercholesterolemia with the annual cognitive score decline rate. We estimated the effect in both unadjusted models and adjusted model with age and APOEε4 genotype. All the coefficients and *p*-values are reported, and the Bonferroni corrected significance level given multiple testing type 1 error reaches *p* = 0.0125 (the significant threshold) in the reported results.

## 3. Results

### 3.1. More MCI Subjects with Early Hypertension Converts to AD than Those Without Early Hypertension

A total of 3052 MCI patients were included in this study (Figure 1). The average age was 87.79 +/− 9.64 (standard deviation (SD)). Among them, 48.26% were male. There were 77.49% White, 16.15% Black, 2.59% Asian, and 3.77% other patients. Out of 3052 subjects, 4.69% carried the APOEε4/4 double allele, and 27.65% carried APOEε3/4 allele (Table 1). In this cohort, 59.07% of patients reported hypertension, and 55.46% of patients reported having hypercholesterolemia before the end of the study (Table 1).

After excluding unqualified patients, there were 906 patients with MCI: 35 of them reported early hypertension, and 18 (51.43%) of them developed AD at follow-up. While the majority of subjects with MCI were at advanced age, >600 subjects with MCI were younger than 80 years old (Supplement Table S1), and 35 subjects reported hypertension within 18 months after the diagnosis of MCI (Table 2). The average conversion time from MCI to AD was 8.09 (±5.95) years (±SD). Among MCI subjects without early hypertension (n = 871), 233 (26.75%) of them developed AD at follow-up. The average conversion time from MCI to AD was 10.30 (±4.99) years (±SD). There was a significant association between early hypertension and AD onset (*p* = 0.0014) (Table 2).

### 3.2. Hazard Ratio of Early Hypertension Is Similar to That of APOEε4 as a Risk Factor of MCI Conversion to AD

Early hypertension has a significantly higher risk and HR of AD onset at follow-up using unadjusted and adjusted Cox models: HR = 2.56, 95% CI (1.55, 4.22), *p* = 0.0002, and HR = 2.77, 95% CI (1.66, 4.65), *p* < 0.0001, respectively. The HR was higher than for those carrying APOEε4 allele (HR = 2.30, 95% CI (1.75, 3.02, *p* < 0.0001)) (Table 3). Similarly, we found a significant association of early hypertension with AD onset with a doubly robust propensity score weighted Cox model (HR = 3.71, 95% CI (3.02, 4.56), *p*-value < 0.0001). The HR was higher than for those carrying the APOEε4 allele (HR = 1.70, 95% CI (1.40, 2.07, *p* < 0.0001)) (Table 3).

We further examined the interaction of APOEε4 and early hypertension on AD onset. Compared to those without APOEε4 and early hypertension, patients with early hypertension but no APOEε4 allele had a significantly higher risk of AD onset (HR = 3.25, 95%CI (1.75, 6.04), *p* = 0.0001), and the patients with APOEε4 (without early hypertension) had a significantly higher risk of AD onset (HR = 2.37, 95% CI (1.79, 3.14), *p* < 0.0001). The HR of APOEε4 (2.37) was lower than early hypertension (3.25). Those with both APOEε4 and early hypertension had the highest risk of AD onset (HR = 4.88, 95% CI (1.93, 12.33), *p*-value = 0.001 (Table 3).

**Figure 2:**
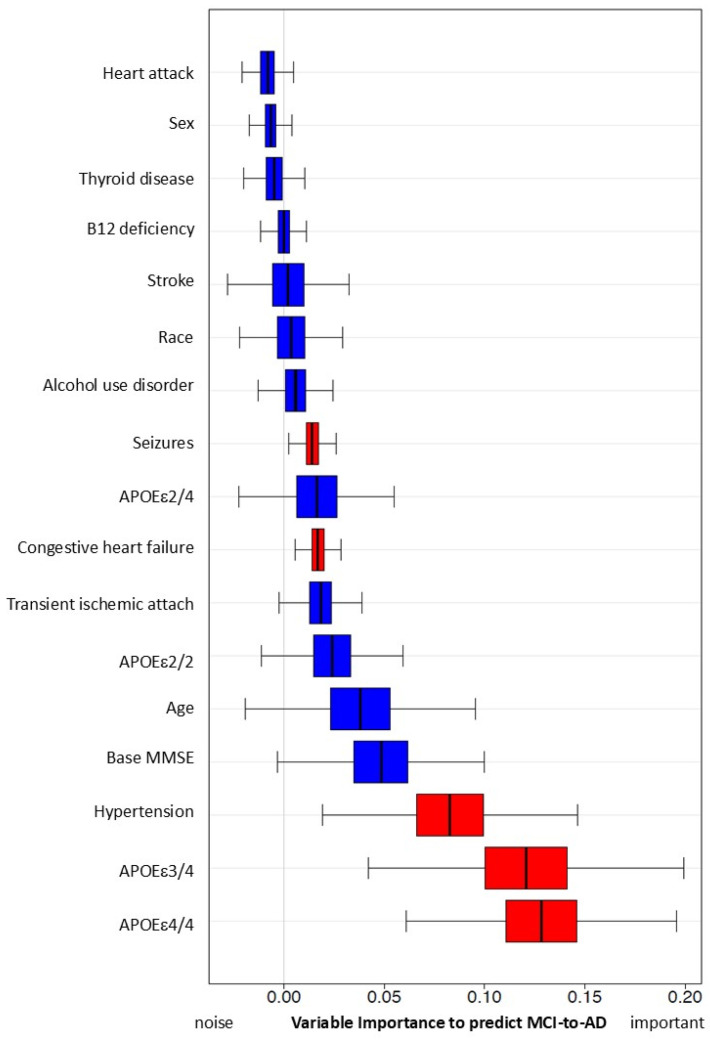
Predictive capacity of hypertension and other risk factors for MCI progression to AD using simulation with Random Forest Method. Variables with red color are significant in this simulation.

To enable separate comparison of MCI subjects with APOEε2/4, ε3/4 and ε4/4, we used a simulation with the Survival Random Forest method (Figure 2). Compared to those with early hypertension, we found that APOEε3/4 and APOEε4/4 carriers had a higher risk of converting MCI to AD, while APOEε2/4 carriers showed no significant difference. Early hypertension was consistently ranked as the most significant risk factor among non-genetic factors, similar to the genetic risk factor of the APOEε4 allele.

Variable importance, calculated from the Survival Random Forest model indicates high predictive ability of each variable (e.g., hypertension). Hypertension has significant variable importance, similar to that of APOEε4/4 and 3/4, and predicts conversion from MCI to AD. Both seizure and congestive heart failure also have statistically significant variable importance/predictive capacity (in red). All other variables (in blue) do not reach significance.

### 3.3. MCI Subjects with Early Hypertension or APOEε4 Had Similar Declining Rates of MMSE Scores

When examining the rate of MMSE score decline among 74 MCI patients, it was found that the 34 subjects having early hypertension showed an annual declining rate of 0.97, compared to those without early hypertension at a rate of 0.38 per year (Table 4). These rates were comparable to those carrying the APOEε4 allele, where APOEε4 carriers showed an annual declining rate of 1.03, compared to those without APOEε4 at a rate of 0.50 per year (Table 4).

Using a Poisson model with the subject’s monitor period as the offset, we estimated the difference in the MMSE score decline rate between subjects with early hypertension and without early hypertension. The model results showed that the annual score declining rate for subjects with early hypertension are expected to be from 2.43 (adjusted) to 4.52 (unadjusted) times the decline rate of non-early hypertension subjects (95% CI (1.33, 4.46), *p* value = 0.004, adjusted; 95% CI (2.47, 8.28), *p* < 0.0001, unadjusted), after controlling for age and APOEε4 (Table 5).

### 3.4. Early Hypercholesterolemia Carries Less Risk Compared to Hypertension or APOEε4

To determine whether hypertension was a unique risk factor among all comorbidities, similar data analysis and statistical models were applied to examine the association of early hypercholesterolemia with the risk of AD onset among MCI patients. We found 75 patients with early hypercholesterolemia and 935 patients without hypercholesterolemia among 1010 qualified patients (Supplement Table S2A,B).

**Figure 3:**
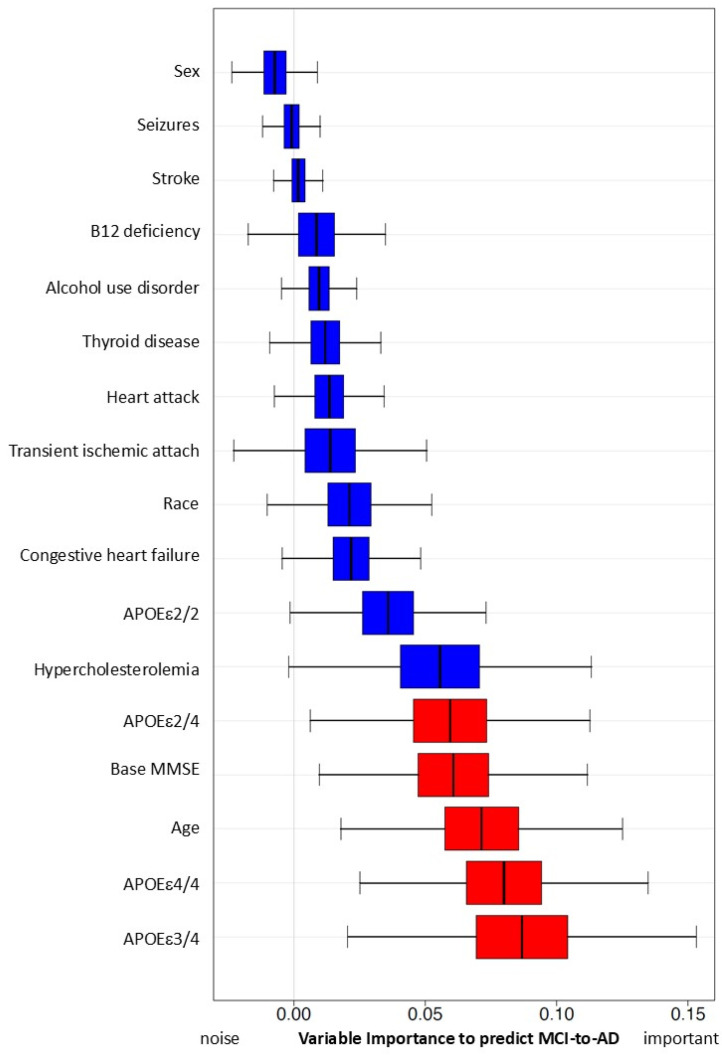
Predictive capacity of hypercholesterolemia and other risk factors for MCI progression to AD using simulation with Random Forest Method. Variables with red color are significant in this simulation.

With all four models (Table 6), we found significant associations between early hypercholesterolemia and AD onset. The adjusted model showed HR = 3.60, 95% CI (2.55, 5.10), *p*-value < 0.001. However, the impact of early hypercholesterolemia on risk of AD onset results are mixed. There are no significant results in our Random Forest simulations (Figure 3).

Variable importance, calculated from the Survival Random Forest model, indicates the high predictive ability of each variable (APOEε4, age, and MMSE score at baseline, in red). Hypercholesterolemia and all other variables (in blue) do not reach statistically significant variable importance to predict conversion from MCI to AD.

In the sub analysis of subjects with APOEε4 and/or early hypercholesterolemia, we find that patients with APOEε4 and early hypercholesterolemia have a higher risk of AD onset compared to the patients without early hypercholesterolemia and the APOEε4 allele (Table 6). In the MMSE sub analysis, after controlling for age and APOE, we did not find a significant impact of early hypercholesterolemia on the annual score decline rate (0.77−0.79/year) (Table 7).

Using a Poisson model, we determined the importance of early hypercholesterolemia by estimating the difference in the MMSE score decline rate between subjects with or without early hypercholesterolemia (Table 8). We found that the annual score declining rate for subjects with early hypercholesterolemia was expected to be from 2.05 (adjusted) to 1.19 (unadjusted) times the decline rate of non-early hypercholesterolemia subjects (95% CI (1.33, 3.20), *p* value = 0.002, adjusted; 95% CI (0.77, 1.84), *p* = 0.44, unadjusted), after controlling for age and APOEε4 (Table 8). In sum, these results indicate that there is no consistent evidence to support the association of early hypercholesterolemia with the annual cognitive score declining rate of MMSE.

## 4. Discussion

In this study, we found a significant association between early hypertension and AD onset among MCI patients. In a sub-analysis with a smaller sample size, we also found a significant interaction between early hypertension and the APOEε4 genotype. Furthermore, we observed a significant acceleration in the decline of MMSE cognitive scores associated with early hypertension. Our findings demonstrate a comparable impact of early hypertension and APOEε4 allele on MCI to AD conversion, indicating potential clinical implication for the healthcare of MCI patients.

In a previous study of 837 MCI patients enrolled at baseline and followed for 5 years, hypertension was associated with an increased risk of progression from MCI to AD [16]. A meta-analysis reviewed 3426 publications, where 7 were eligible studies. These studies provide a comprehensive overview of the relationship between hypertension and AD risk, and significant associations between hypertension and AD onset [19]. However, the influence of the timing of hypertension after MCI diagnosis with controls for APOE genotype has not been studied. In this study, we focused on the impact of early hypertension on AD onset, which is defined as the first hypertension diagnosis within 18 months after the first MCI diagnosis.

The role of APOE in AD is well established, and APOEε4 is the strongest genetic risk factor for AD [14]. Controlling for the age factor and APOE genotype, early hypertension is significantly associated with an increasing risk of AD onset and MMSE score with an annual decline rate. However, the impact of early hypercholesterolemia is not consistent. On the other hand, early hypertension is a unique chronic condition that is a strong risk factor for MCI converting to AD.

Our study offers valuable insights into the progression and risk factors of AD, but several limitations should be noted. First, hypertension and hypercholesterolemia require patients to take medications on a daily basis, but we do not have information on the duration and dosage of these medications, which may potentially influence the outcomes. Previously, we reported the beneficial association of statin use on the occurrence of possible AD, which is critical in assessing the contribution of hypercholesterolemia to the development of AD [11]. Since these medications are used on a daily basis, the use of self-reported comorbidities without a clinical diagnosis date should be fairly accurate in the NACC database. We do note that self-reported data are susceptible to various biases, such as reporting biases which might affect the accuracy of the information collected. However, self-reported measures has been shown to be reliable from the past published literature [20]. The limitation of the lack of data on the initiating time of prescriptions is important for understanding the temporal relationships between drug exposure and AD onset. The absence of dosage information further complicates this issue, as it prevents us from assessing the potential dose–response relationship, which may be of consequence for establishing the efficacy of medications in relation to AD.

Second, the lack of the Montreal Cognitive Assessment (MoCA) scores [11] to cross-validate the MMSE scores is another limitation. The MoCA is considered more sensitive than the MMSE for detecting cognitive impairment [21], and its absence could potentially affect the study’s conclusions regarding the progression from MCI to AD with missed detections of cognitive impairment. Lastly, the small sample size for APOEε4 carriers with MMSE test scores might impact the results and limit the generalizability of the findings. However, based on our group size and event rates, we have a medium effect size from the power analysis with 80% power at the 0.05 significance level. Therefore, future research should extend this analysis to a much larger database, such as the administrative and clinical data from the Veterans Affairs Healthcare System, which contains both detailed medication prescription data in the context of a longitudinal time frame over decades.

Studies of early hypertension are important for elders, especially patients with MCI. The relationship between hypertension and the risk of hemorrhagic events has garnered increasing attention with MCI and AD. A study in 2013 called for more novel therapies for intracerebral hemorrhage (ICH), since it is considered as the most severe form of stroke [22]. Recent research has found that AD patients have a higher risk of hemorrhagic but not ischemic stroke compared with non-AD controls with similar risk profiles [23]. Another study in 2017 found that one of the main risk factors for ICH is hypertension [24]. The increased risk of hemorrhagic stroke in patients with AD could be driven by the association of AD with cerebral amyloid angiopathy (CAA), with thinning of the endothelial cytoplasm, loss of pericytes, and endothelial proteins of the blood-brain barrier [25,26]. Hypertension-related ICH arises from chronic vascular stress, affecting deeper brain structures such as the basal ganglia, thalamus, and brainstem rather than the cerebral cortex [27]. When MCI or mild AD patients are vulnerable to hemorrhage, newly FDA-approved anti-amyloid therapy needs to be carefully evaluated for those patients due to the side effect of micro hemorrhage and edema [28]. Whether these patients have a comorbidity of hypertension is one of the critical factors to determine the use of anti-amyloid therapy, which is similar to the APOE genotype [29]. AD patients with two APOEε4 alleles have a much higher risk of experiencing micro-hemorrhage and edema [28], and our findings highlight a second risk factor of hypertension when considering anti-amyloid therapy. Therefore, more studies of hypertension’s impact on the disease progression from MCI to AD are needed, and early hypertension as a modifiable risk factor should be extensively studied and managed in clinical care.

## Figures and Tables

**Table 1:** Demographic information of MCI patients.

	MCI Patients	
	N = 3052	
	**Mean**	**SD**
Age	87.79	9.64
Base MMSE	27.02	2.47
	**N**	**%**
Male	1473	48.26
Female	1579	51.74
Race (white)	2365	77.49
Race (Black)	493	16.15
Race (Asian)	79	2.59
Race (Others)	115	3.77
APOE Genotype		
APOEε4/4	143	4.69
APOEε3/4	721	27.65
APOEε4/2	50	1.92
APOEε3/3	1153	44.21
APOEε3/2	203	7.78
APOEε2/2	10	0.38
APOE not available	772	29.60
Comorbidities		
Hypercholesterolemia	1671	55.46
Hypertension	1795	59.07
Thyroid Disease	513	16.92
Diabetes	507	16.68
Heart Attack/Cardiac Arrest	246	8.08
Stroke	267	8.80
Transient Ischemic Attack	202	6.70
Alcohol Abuse	184	6.04
B12 Deficiency	129	4.32
Congestive Heart Failure	107	3.51
Seizures	111	3.65

**Table 2:** Conversion time from MCI to AD.

Conditions	Number of MCI Subjects at Index	Number of MCI-to-AD Subjects at Study End	%	MCI Conversion Time to AD (Year) (±SD)
MCI with early hypertension	35	18	51.43	8.09 (5.95)
MCI without early hypertension	871	233	26.75	10.30 (4.99)

**Table 3:** Comparing the impact of post-MCI hypertension and APOEε4 by unadjusted, adjusted, propensity score weighted (PSW), and interaction Cox models.

Models	Variables	HR	*p*-Value	95% CI	
Unadjusted Cox model	MCI with hypertension vs. MCI without hypertension	2.56	0.0002	1.55	4.22
Adjusted Cox model	MCI with hypertension vs. MCI without hypertension	2.77	<0.0001	1.66	4.65
	APOEε4	2.30	<0.0001	1.75	3.02
PSW doubly robust Cox model (no APOEε4/Early Hypertension as reference)	Early hypertension	3.71	<0.0001	3.02	4.56
	APOEε4	1.70	<0.0001	1.40	2.07
Adjusted Interaction Cox model (no APOEε4/Early Hypertension as reference)	Early Hypertension	3.25	0.0001	1.75	6.04
	APOEε4	2.37	<0.0001	1.79	3.14
	APOEε4 + Early Hypertension	4.88	0.001	1.93	12.33

Note: 1. For the PSW double robust model, propensity score was created to balance early hypertension and non-early hypertension group based on age, sex, and APOEε4. 2. All adjusted models are adjusted by age, sex, APOEε4, race, and comorbidities.

**Table 4:** Decline rate of MMSE scores among MCI subjects.

	N	Mean	Std.
No early hypertension	40	0.38	0.50
Early hypertension	34	0.97	1.40
No APOEε4	52	0.50	0.85
APOEε4	22	1.03	1.39

Note: MMSE score decline rate = (max MMSE score − last MMSE score)/duration. The last MMSE score is also the most recent MMSE score for the patient in our record.

**Table 5:** Association of hypertension with the MMSE score decline among MCI subjects using Poisson model.

Poisson Model	Variables	B	Standard Error	95% CI		*p*-Value	exp^B	95% CI of exp^B	
Unadjusted	Intercept	−2.92	0.25	−3.42	−2.42	<0.0001	0.05	0.03	0.09
	early hypertension vs. none	1.51	0.31	0.90	2.11	<0.0001	4.52	2.47	8.28
Adjusted	Intercept	−1.19	0.28	−1.75	−0.64	<0.0001	0.30	0.17	0.53
	early hypertension vs. none	0.89	0.31	0.28	1.50	0.004	2.43	1.33	4.46

Note: “B” is the coefficient of variables from the model. Since log transformation is used in Poisson model on the MMSE score decline rate ratio, we interpret exp^B instead of B. MCI subjects with early hypertension have decline rates 4.52 and 2.43 times of those without early hypertension, using unadjusted and adjusted models, respectively.

**Table 6:** Comparing post-MCI early hypercholesterolemia and APOEε4 using unadjusted, adjusted, PSW adjusted, and interaction Cox models.

Models	Variables	Coefficient	Std. Err.	*p*-Value	HR	95% CI	
Unadjusted	Early hypercholesterolemia	1.48	0.16	<0.0001	4.38	3.15	6.07
Adjusted	Early hypercholesterolemia	1.28	0.17	<0.0001	3.60	2.55	5.10
	APOEε4	0.66	0.14	<0.0001	1.93	1.46	2.55
PSW adjusted	Early hypercholesterolemia	1.35	0.09	<0.0001	3.88	3.26	4.61
	APOEε4	1.31	0.23	<0.0001	3.70	2.34	5.83
Adjusted Interaction	APOEε4	0.67	0.16	<0.0001	1.95	1.43	2.67
	APOEε4 and early hypercholesterolemia	1.92	0.25	<0.0001	6.82	4.16	11.18

Note: Subjects without early hypercholesterolemia and APOEε4 are the reference group.

**Table 7:** MMSE score decline rates among subjects with or without early hypercholesterolemia or APOEε4.

	N	Mean	Standard Deviation
No early hypercholesterolemia	51	0.79	0.89
Early hypercholesterolemia	51	0.77	1.09
No APOEε4	64	0.54	0.66
APOEε4	38	1.19	1.28

Note: MMSE score decline rate = (max MMSE score − last MMSE score)/duration. The last MMSE score is also the most recent MMSE score in the record.

**Table 8:** Association of hypercholesterolemia with the MMSE score decline among MCI subjects, using Poisson model.

Poisson Model	Variables	B	Standard Error	95% CI		*p*-Value	exp^B	95% CI of exp^B	
Unadjusted	Intercept	−1.92	0.16	−2.23	−1.61	<0.0001	0.15	0.11	0.20
	Early hypercholesterolemia vs. none	0.17	0.22	−0.27	0.61	0.44	1.19	0.77	1.84
Adjusted	Intercept	−2.23	0.20	−2.61	−1.84	<0.0001	0.11	0.07	0.16
	Early hypercholesterolemia vs. none	0.72	0.23	0.27	1.16	0.002	2.05	1.31	3.20

Note: “B” is the coefficient of variables from the model. MCI subjects with early hypercholesterolemia have a decline rate 1.19 (insignificant) and 2.05 (significant) times of those without early hypercholesterolemia, using unadjusted and adjusted models, respectively.

## Data Availability

The original contributions presented in this study are included in the article/Supplementary Material. Further inquiries can be directed to the corresponding author(s).

## Supplementary Materials

The following supporting information can be downloaded at: https://www.mdpi.com/article/10.3390/brainsci15050434/s1, Table S1: Distribution of patients with MCI by age; Table S2A: Conversion time from MCI to AD among subjects with or without early hypercholesterolemia; Table S2B: Percentage of subjects with or without early hypercholesterolemia converting from MCI to AD.

## Abbreviations

The following abbreviations are used in this manuscript:

AD	Alzheimer’s disease
APOE	apolipoprotein E
CDC	Centers for Disease Control and Prevention
HR	Hazard Ratio
MCI	Mild Cognitive Impairment
MMSE	Mini-Mental State Examination
MoCA	Montreal Cognitive Assessment
NACC	National Alzheimer’s Coordinating Center
PSW	propensity score weighted
SD	standard deviation
