# Supplementary material for: Hypertension a Predictive Risk Factor on Progression to Alzheimer’s Disease Using APOEε4 as a Benchmark
Source: Brain Sci. 2025 Apr 23;15(5):434. doi: 10.3390/brainsci15050434 (PMC12110230; doi:10.3390/brainsci15050434)
Supplement: Supplementary file 1 [file brainsci-15-00434-s001.zip › brainsci-3585091-supplementary.pdf]

Supplementary Materials

Table S1
Distribution of patients with MCI by age.

Age	MCI	MCI-to-AD
65-70	116	21
70-75	192	51
75-80	301	109
80-85	486	197
85-90	567	273
90+	1390	697

Table S2A
Conversion time from MCI to AD among subjects with or without early hypercholesterolemia.

	Early hypercholesterolemia			No early hypercholesterolemia			
Total Subject number	Subject number	Mean conversion time (years)	Standard Deviation	Subject number	Mean conversion time (years)	Standard Deviation	p-value
1010	75	7.15	5.47	935	11.07	4.75	<0.0001

Table S2B
Percentage of subjects with or without early hypercholesterolemia converting from MCI to AD.

	Early hypercholesterol-emia		No early hypercholesterol-emia		p-value
	N	AD/MCI %	N	AD/MCI %	
MCI	75		935		
MCI-to-AD	48	64.00	198	21.18	<0.0001
